# Annotations

**Published:** 1908-05-30

**Authors:** 


### Annotations.

### Tuberculous Meat.

It is matter for concern to many besides those who dwell in the City of London itself that Dr. Collingridge can find so much that is unsatisfactory about the produce vended in Smithfield Market. In his annual report, lately published, the medical officer of health says that " although the falling off in the quantity " (of meat) " actually condemned as unfit for human food is satisfactory as showing some improvement in methods of transit, yet it could hardly be contended that the quality of meat on which the population of London lives is not making retrograde movements year by year." More disquieting still are the facts cited which show how little reliance can be placed on the certificates of some veterinary surgeons, and even county veterinary inspectors seem sometimes to be guilty of culpable negligence. The inspection label of the United States Department of Agriculture appears to be no more an infallible guarantee of the soundness of that which bears it, for details are given of carcasses bearing it which, although cold stored, were clearly unfit for human consumption, owing to pre-existing disease. In one case a farmer sent up a consignment of pigs, accompanied by a certificate of freedom from disease, but six of them turned out to be tuberculous. To call the attention of the public, both medical and lay, to facts and figures of the kind furnished in this report is not an unnecessary scare-mongering, but a very real obligation. How long the British people must be stimulated with an occasional prod of inconvenient fact before it will take efficient action to protect itself from horrors of this kind it is difficult to say; but from the signs of the times there seems to be a growing tendency towards wakefulness, and not any too soon. We could wish that Dr. Collingridge's report might be as widely studied as it deserves, for then there might also be reasonable hope of these tuberculous meat scandals coming to an end.

### International Cancer Research.

On May 23 an International Association for Cancer Research was inaugurated at Berlin. The objects of the Association is to promote the investigation of cancer and the cure of persons afflicted with cancer, and to disseminate knowledge of this disease. It is proposed to collect and publish cancer statistics from all countries, and to establish an international centre of information on all matters concerning cancer research. An endeavour is being made by the Association to introduce a uniform system of statistics. The central office will be situated at Berlin, and from this will be published an international journal of cancer research, while an important part of the work of the Association will be the organisation of international cancer conferences. A large number of the leading members of the German medical profession were present at the opening ceremony at Berlin to welcome the representatives of foreign committees. Professor von Leyden and Dr. Holle, of Berlin, addressed the assembly; and papers were read by Professor Borel, of Paris; Professor Dollinger, of Budapest; Professor Jensen, of Copenhagen; and Professor Podvyssotzki, of St. Petersburg. At the time of the first meeting it was announced that thirteen States had already joined the Association, including all the great Powers with the exception of Great Britain. The formation of the International Cancer Research Association is largely due to the energy of Professors Czerny, von Leyden, Ehrlich, and Orth, who are all members of the German Central Committee. Much enthusiasm was expressed by the various representatives at the inaugural meeting. All were agreed that the time has come for concerted action by the many workers scattered throughout the civilised world. The new Association has undertaken the task of organisation, without which combined action is impossible. If this work is well done at the headquarters in Berlin, individual enterprise should receive a stimulus, and a judicious blending of co-operation and healthy rivalry should advance the cause of cancer investigation.

### Medical Reciprocity with Canada.

At the commencement of the eighty-seventh session of the General Medical Council, on May 26, Dr. Donald Macalister, the president, in his opening address, said that an order had recently been issued by the Privy Council, recognizing the Province of Quebec as a separate British possession, and applying to it the provisions of Part II. of the Medical Act, 1886. In pursuance of the Order in Council, two Universities, namely, the McGill University of Montreal and the Laval University of Quebec, had submitted to the executive committee particulars of their medical curricula and examinations, with a view to the recognition of their degrees for the purpose of registration. The committee had satisfied itself that the degree of M.D. granted by these important bodies furnished a sufficient guarantee of the proficiency of their graduates, and in accordance with the council's standing order, had agreed that they should be recognised under the conditions prescribed in the Medical Act. The War Office authorities were prepared to offer to Quebec graduates, registered in this country, the same facilities in respect of commissions in the Army medical service as were enjoyed by registered graduates from Nova Scotia. It was to be hoped that the recognition accorded to the two provinces might have the effect of inducing the other provinces of the Dominion to reconsider the question of reciprocity from a national, rather than a local point of view. At the conclusion of the first day's sitting a resolution was passed recording the Council's satisfaction at the establishment of reciprocal relations with the province of Quebec, and its desire that similar relations might soon be established with other provinces in Canada. This is a view which we think will be endorsed by the great majority of members of the medical profession in Great Britain and throughout the Empire. There is much to be gained and little to be lost by a complete system of imperial medical reciprocity and co-operation.

